# A Case of Pseudomembranous Colitis of Unknown Etiology

**DOI:** 10.7759/cureus.21914

**Published:** 2022-02-04

**Authors:** Ariana R Tagliaferri, Kelsey Murray, Patrick Michael

**Affiliations:** 1 Internal Medicine, St. Joseph’s University Medical Center, Paterson, USA; 2 Medical Student, St. Joseph’s University Medical Center, Paterson, USA

**Keywords:** pseudomembranous colitis, enterocolitis, diarrhea, clostridium infections, clostridium difficile

## Abstract

Pseudomembranous colitis (PC) is a nonspecific bowel injury resulting from decreased oxygenation, endothelial damage, and impaired blood flow to the mucosa. Although the most well-known cause of PC is *Clostridium difficile (C. difficile)*, several diseases and medications can cause or predispose individuals to PC, such as microscopic colitis, infectious organisms, inflammatory conditions, nonsteroidal anti-inflammatory drugs, and chemotherapy agents. Here, we present the case of a patient who completed treatment for *C. difficile* infection but developed worsening PC of unknown etiology.

## Introduction

Hospitalized patients on antibiotics are at an increased risk of acquiring nosocomial diarrhea because antibiotics can disrupt their normal gut microflora [1,2]. Nosocomial diarrhea is most commonly caused by *Clostridium difficile* (*C. difficile*), a spore-forming, obligate, anaerobic, gram-positive bacillus [1]. The virulence of *C. difficile* increases due to its ability to produce toxins A and B [2]. Clinically, *C. difficile* is treated with metronidazole and oral vancomycin; however, in recurrent cases, clinical failure, or antibiotic resistance, clinicians may seek other non-antibiotic therapeutics, such as fecal transplant, which reintroduces healthy diverse bacteria to reestablish bacterial colonization [2,3]. Failure of initial antibiotic therapy occurs in 20-30% of cases, with 40-60% of patients having a recurrent episode [1]. This not only increases morbidity for the patient but is a major financial burden on the healthcare system [1]. In cases where *C. difficile* is refractory to treatment, clinicians should look for other causes of diarrhea.

*C. difficile* and pseudomembranous colitis (PC) are not interchangeable terms. PC is a nonspecific bowel injury resulting from decreased oxygenation, endothelial damage, and impaired blood flow to the mucosa [4]. A pseudomembrane is a layer of fibro-purulent exudate composed of inflammatory cells and mucus originating from inflamed and erupting crypts and has various etiologies [4]. Although the most well-known cause of PC is *C. difficile*, several diseases and medications can cause or predispose individuals to PC [4], such as microscopic colitis, infectious organisms, inflammatory conditions, nonsteroidal anti-inflammatory drugs, and chemotherapy agents [4]. The most common medications include docetaxel, 5-fluorouracil, cyclosporine, cisplatin, diclofenac, and indomethacin [4]. The most common infectious etiologies include *Staphylococcus aureus*, *Escherichia coli*, cytomegalovirus, and cryptosporidium [4]. Thus, because there are many etiologies of PC, clinicians should be open-minded and pursue full workup in the setting of *C. difficile*-negative diarrhea with diagnostic evidence of colitis.

## Case presentation

A 73-year-old Hispanic male, with a medical history of recent cholecystostomy and subsequent cholecystectomy, presented to the Emergency Department (ED) with subacute worsening diarrhea refractory to antibiotics. The patient had initially presented to an outside hospital three months prior with abdominal pain and was admitted to the surgical team for acute cholecystitis. At that time, a Blake drain was placed for inflammation, and the patient was scheduled for elective cholecystectomy three months later. During this time, the patient developed large voluminous bowel movements approximately three to four times a day. He then underwent an uncomplicated robotic laparoscopic cholecystectomy but developed worsening diarrhea on postoperative day (POD) four. He then tested positive for *C. difficile* via polymerase chain reaction (PCR) and completed a 10-day course of metronidazole 500 milligrams every eight hours and vancomycin 500 milligrams twice daily. Although he had completed the course of antibiotics the day before the admission, he was experiencing more than 12 watery bowel movements daily, now with more than two tablespoons of blood mixed with the stool, prompting him to present for further evaluation. The stool was light brown to yellow colored and associated with urgency. He denied fecal incontinence, mucus, nausea, vomiting, and abdominal or rectal pain with defecation. He also denied recent travel, sick contacts, changes in diet, or the use of dietary supplements. A review of the systems was positive for decreased appetite and unintentional weight loss of approximately 30 pounds within the last month, as well as generalized fatigue and weakness.

**Figure 1:**
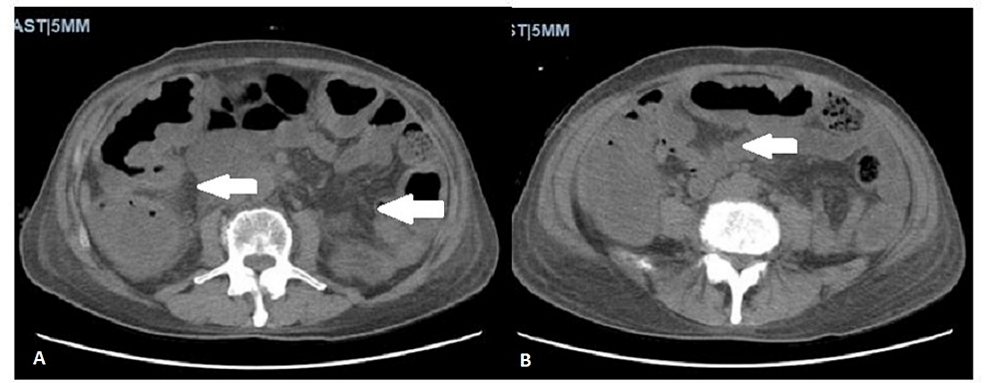
Computerized tomography of the abdomen and pelvis without contrast. Two axial images demonstrating severe colitis with peri-colonic fluid (white arrows), representing pseudomembranous enterocolitis worse from the previous study. No appendicitis, diverticulitis, or bowel obstruction was noted.

On admission, the patient was afebrile, tachycardic (101 beats per minute), hypotensive (89/60 mmHg), and saturating 99% on room air. He appeared cachectic with bitemporal wasting and dry mucus membranes but was not in acute distress. He had an abdominal scar in the right mid-axillary line where the Blake tube was inserted previously, without drainage or erythema, and his abdomen was soft and non-tender with normoactive bowel sounds. He demonstrated no skin lesions or lymphadenopathy. On digital rectal examination, the patient had no external/internal lesions or erythema. Moreover, normal sphincter tone and liquid stool mixed with streaky blood were present on the digit upon removal. Labs were remarkable for hyponatremia (127 mEq/L), hypochloremia (95 mEq/L), evidence of malnutrition and dehydration (blood urea nitrogen 8 mg/dL, creatinine 0.6 mg/dL, and total protein 4.1 g/dL), normocytic anemia (hemoglobin 11.6 g/dL, mean corpuscular volume 86.9 fL), and leukopenia (1.8 × 10^9^/L). Fecal calprotectin was elevated (816 μg/g), and *C. difficile* PCR toxin and antigen were negative. All other labs, including human immunodeficiency virus, urinalysis, thyroid function, methicillin-resistant *Staphylococcus aureus* PCR, and procalcitonin were unremarkable. Computerized tomography (CT) of the abdomen and pelvis without contrast showed worsening pseudomembranous enterocolitis from previous admission (Figure 1).

**Figure 2:**
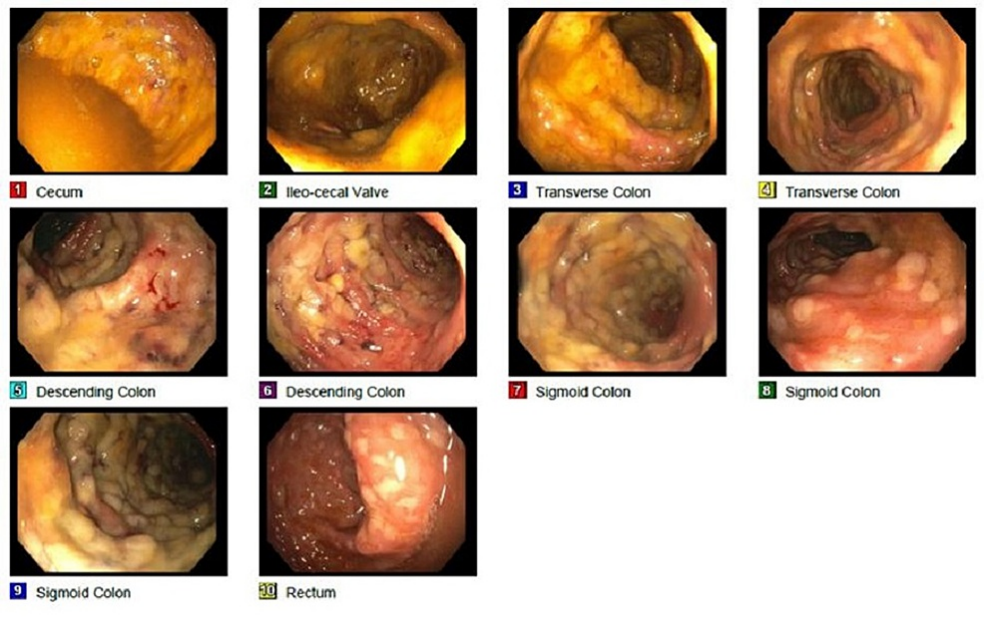
Colonoscopy demonstrating pseudomembranous colitis. Perianal and digital rectal examinations were unremarkable. Diffuse and severe pseudomembranes with friability, erythematous, and inflammatory changes were seen in the entire colon, more prominent in the left colon. Continuous ulcerated areas covered with fibrinous exudate are seen best in images 6-10.

At this stage, the patient became more hypotensive with leukocytosis (15.3 × 109/L) and was transferred to the Intensive Care Unit with a working diagnosis of fulminant *C. difficile*. He was treated with the same regimen of oral vancomycin and intravenous metronidazole with additional vancomycin 500 mg enemas. Because the patient never required pressors, he was transferred back to the medical floor and underwent colonoscopy, which demonstrated diffuse yellowish, whitish plaques with fibrinous exudate consistent with classic PC (Figure 2).

**Figure 3:**
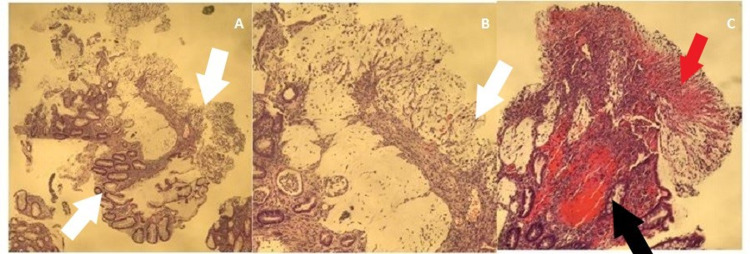
Pathology report obtained from colonoscopy biopsy. Three pathology images demonstrating pseudomembranous layers of fibro-purulent exudate (red arrow) and mucus with erupting “volcanic” crypts (black arrow) and loss of cryptic architecture (white arrows), consistent with pseudomembranous colitis.

Repeat testing for *C. difficile* along with blood cultures, stool cultures, and ova and parasites were negative. The pathology report revealed layers of fibro-purulent exudate and mucus with erupting “volcanic” crypts and loss of cryptic architecture (Figure 3).

The patient ultimately improved after receiving a two-day course of fidaxomicin. All other antibiotics were stopped. He was medically optimized and discharged to complete a total 10-day course of fidaxomicin.

## Discussion

PC presents with fever, abdominal pain, leukocytosis, diarrhea, toxic megacolon, and electrolyte imbalance [4]. Clinicians should pursue further workup when colitis is seen on endoscopy but *C. difficile* testing is negative, and/or if the patient is refractory to treatment. *C. difficile* PCR has a sensitivity and specificity of 97% and 93%, respectively [4]. If PCR is negative, repeat testing is unnecessary. Regardless of etiology, PC has the same gross endoscopic appearance of friable and erythematous mucosa with ulcerative lesions covered in fibrinous exudate [3,4]. The pathognomonic term to describe PC is a volcanic eruption of fibrin and pus, which is evident on macroscopic and microscopic appearance, regardless of the cause [4]. However, there are microscopic pathological findings specific to the etiology, such as acute crypt injury and dilation involving the upper lamina propria and crypts filled with exudate seen with *C. difficile*-induced colitis versus additional findings of cell apoptosis and increased intraepithelial lymphocytes seen in drug-induced colitis [4]. Neither of these pathological subsets was visualized on the biopsy taken from our patient, in which only evidence of PC was detected without other specific findings. Because our patient was in septic shock during hospitalization, it can be questioned whether he developed superimposed, ischemia-induced colitis. However, on microscopic visualization, one would detect hyalinization of the lamina propria, hemorrhage, and full-thickness mucosal necrosis, none of which were seen on our patient’s pathology slides.

Although biopsy can help diagnose and direct management in cases where the etiology is unclear, our patient’s case was more complex without definitive answers.

## Conclusions

The management of nosocomial diarrhea can be complex and warrants numerous investigations for appropriate treatment. However, because it is difficult to determine which situations require an extensive workup, clinicians should pursue further workup when colitis is evident on diagnostic imaging or endoscopy, if *C. difficile* testing is negative, and/or if the patient is refractory to treatment. It is important to remember that PC and *C. difficile* are not interchangeable terms, and biopsy can be helpful in differentiating unclear etiologies of colitis. If left untreated, diarrhea can lead to a significant healthcare burden and, more importantly, patient demise.
